# News: Resources

**Published:** 2011-06

## Environment

### Study reveals global hotspots of climate change and food insecurity

A project by the CGIAR Research Program on Climate Change, Agriculture and Food Security (CCAFS) was set out to identify hotspots of climate change and food insecurity. They reported nine climate change indicators which included decline in the length of the growing period for crops, long periods of high temperature and increase in rain intensity. When these were combined, Southern Africa emerged as highly exposed, followed by the regions of northeast Brazil, Mexico, Pakistan, India and Afghanistan. This analysis means that millions of people already living in poverty are to be challenged further, with hotter and more variable weather, which will pose a threat to crop yields and livestock.

### Strange mass death events affecting animal species are being reported worldwide

Since the middle of 2010, the world media have been reporting a series of rather strange events involving the mass death of different animal species. Birds and fish were predominantly involved. The events seem to be isolated from each other, but they have received a significant attention from the media and internet bloggers. The academics are still uncertain on how to explain these rare events. There is a growing list of proposed factors and explanations, but very little certainty over the true causes. Some believe that these mass death events are truly alarming, because they might indicate an early warning about the disturbances in Earth’s natural cycle, which could possibly be related to the global climate change. Among suspected or proposed causes, the media suggested new infectious diseases, earthquakes or mass collisions and stress caused by fireworks.

### Australia experiences some of the worst floods in living memory

A high intensity rainfall in January 2011 caused major flooding across much of the western and central parts of the state of Victoria, Australia. Although the true extent of the damage is nearly impossible to assess, a rough estimate predicted a loss in revenues from Australia’s GDP of nearly 30 billion Australian dollars. The floods damaged a large portion of Australia’s coal mines and cotton fields, along with many other natural resources. The events follow the 2010 weather pattern of La Nińa, which brings wetter conditions to eastern Australia. It has been reported that last year’s La Nińa was the strongest since 1973.

### A book suggests links between climate change, health and political stability

Dan Ferber and Paul Epstein are the authors of the new book: Changing Planet, Changing Health: How the Climate Crisis Threatens Our Health and What We Can Do About It (University of California Press, April 2011). According to the authors, climate change threatens more than our environment. Steadily rising temperatures have already led to the spread of infectious diseases – such as malaria in Kenya, Lyme disease in Maine, and cholera. It is also thought to contribute to food shortages and malnutrition. An unstable climate can even fuel political and social unrest – for which they see an example in the recent revolts in the Middle East and North Africa.

### A report points to high corruption levels in countries most affected by climate change

The watchdog group Transparency International (TI) has released a report entitled Global Corruption: Climate Change, based on contributions from more than 50 experts. According to this report none of the countries most affected by climate change (mainly in Africa and South Asia) scored higher than 3.5 in a corruption scale, with 0 being extremely corrupt and 10 being very transparent. They stated that corruption risks are high because of the complexity, uncertainty and novelty around many climate issues and mechanisms to fight climate change need to be strengthened and made more transparent to reduce increasing risks of corruption.

## Demography

### More than a billion residents of India to get unique twelve digit identification numbers

‘Aadhar’, a project to provide unique twelve digit identification numbers (UID) to all residents of India, was initiated in January 2009. Apart from providing identity, the UID will enable better delivery of services and effective governance. In becoming a single source of identity verification, it could enable the easier roll-out of wide number of services such as bank accounts, passports, driving licenses and many others. It is hoped that proof of identity and greater financial inclusion could lay the basis for checking fraud and corruption, avoiding duplication and targeting intended beneficiaries in a range of welfare programs. The first set of Aadhar cards were handed over by the Indian Prime Minister in September 2010 and it is expected that in the next five years all 1.2 billion Indian residents will have an Aadhar card.

### Hundreds of millions keep moving to urban areas in China

One of the main strategic focuses of China’s economic plan for the next decade is the idea of urbanisation. In the past 20 years, more than 200 million people have moved from villages to bigger cities, which is the most extensive process of urbanisation the world has ever seen. According to some estimates, urbanisation will continue to grow, with as many as 300 million people moving to cities over the next couple of decades. The country’s leaders hope that urbanisation will transform hundreds of millions of Chinese into consumers. At the same time, the movements to cities should help maintain high investment rates.

### New census exposes gender imbalance in India

India’s 2011 census shows a serious decline in the number of girls under the age of seven, with the female/male ratio dropping from 0.98 (in 1961) to 0.92 (in 2011). This represents the most striking gender imbalance seen since the Indian independence. Although the proportion of women in the Indian population is steadily growing (mainly due to factors such as longer life expectancy), India’s ratio of young girls to boys is one of the most unbalanced in the world. Some researchers explained this by neglect of very young female children and possibly the increased availability of antenatal screening for gender. Activists interested in this issue claim that the current level of imbalance suggests that up to eight million female foetuses may have been aborted in the past decade in India.

### One-child policy in China may be revised in urban areas

China’s one-child policy, which was introduced in 1979, was a major demographic policy decision launched at the beginning of China’s economic reforms. In 2007, Chinese authorities assessed that the policy had prevented about 400 million births. The policy has been revised in the rural and minority areas of China where, if the first child of a family was a girl, the family is allowed to have a second child. On March 6 this year, during the annual Chinese People’s Political Consultative Conference and the National People’s Congress, further revisions have been proposed. Experts have suggested that since the aging population problem has become increasingly prominent, and with the growing sex-ratio imbalance, a revision of one-child policy should also be considered in urban areas, starting from 2015.

### UN reports steady rise of refugees

According to the New York Times, The United Nations refugee agency reported that 43.7 million people around the globe are displaced from their homes by conflict or persecution. The number is the highest reported in the past 15 years. In addition, 80% of all refugees in the world are being sheltered in the world’s poorest countries, which cannot continue to withstand this large burden on their own. The UN’s refugee agency, based in Paris, France, suggested in its 2010 Global Trends report that Pakistan, Iran and Syria were the world’s biggest hosts of refugees, sheltering about three million people. Among the wealthy countries, Germany has the largest refugee population (about 600 000 people). António Guterres, the United Nations high commissioner for refugees, said that “Fears about supposed floods of refugees in industrialized countries are being vastly overblown or mistakenly conflated with issues of migration, with... poorer countries (being) left having to pick up the burden.” He urged industrialized nations to address this worrying imbalance by increasing the number of people they accept.

## Economy

### South Africa becomes the fifth member of the ‘BRIC club’

According to the Guardian, Jacob Zuma, South Africa’s president, had succeeded in gaining an invitation to join the BRIC (now BRICS) club of Brazil, Russia, India and China in their third summit on China’s Hainan Island in April 2011. With the United States and Europe still trying to overcome the 2008 financial crisis, these five large, populous and fast growing economies are trying to challenge the world’s traditional councils of power. Recently, after the announcement that the French Finance Minister Christine Lagarde will be the likely new head of the International Monetary Fund (IMF), the BRICS club stated that the choice of managing director should not be based on nationality alone, but also on competence.

### G8 leaders off track on their Gleneagles commitments

In the G8 summit in Gleneagles two years ago, the leaders of the eight richest countries agreed to double their annual aid to poor nations to US$ 50 billion (€ 58 billion) per year, with half of that money going to the world’s poorest countries in Africa. But during the recent G8 summit in Heiligendamm, it was clear that the rich world was well off track to deliver on their Gleneagles commitments. A report by Oxfam suggested that, if present trends were to continue, the G8 would miss its target by about US$ 30 billion (€ 21 billion).

### One thousand economists join the call on G20 to accept ‘Robin Hood’ (Tobin) tax

The Robin Hood tax is a package of financial transaction taxes. It was proposed by a campaigning group, largely composed of civil society non-governmental organizations. Campaigners have suggested the tax could be implemented globally, regionally or unilaterally by individual nations. Conceptually similar to the Tobin tax (which was proposed on foreign currency exchange only), it could be imposed on the purchase and sale of stocks, bonds, commodities, unit trusts, mutual funds, and derivatives such as futures and options. Recently, a thousand economists from 53 countries have written to G20 finance ministers asking them to apply the ‘Robin Hood tax’ on transactions in financial markets. The idea is to levy a very small charge (around 0.05%) and use the money raised from this charge to maintain rich nations’ commitments to the developing world. One of the main objections to this tax from the speculators is that since there are millions of trades every minute in global financial markets it will be unworkable to calculate the tax. However, this argument is rather unconvincing, since share transactions are already being taxed.

### Brazil seeks a role in Africa through friendly approach towards local workforce

According to Reuters, Brazil seeks a different approach in Africa from that already practiced by China. At building sites from Angola to Zambia, teams of Chinese workers often do the work instead of Africans. Wherever local African residents are employed, there have been reports that Chinese may be treating them rather roughly. But engineering groups from Brazil, such as Odebrecht (recently contracted to fix Liberia’s railway), decided to employ locals and to treat them well. Odebrecht and other Brazilian companies want to distinguish themselves from companies from other emerging powers and to find a sustainable role in Africa through an approach which is friendly to the locals.

### India announces plans to invest in Africa

The 2nd Africa – India Forum Summit was held this year in the Ethiopian capital, Addis Ababa. During the opening session of the summit, Indian Prime Minister Manmohan Singh announced that India will offer to Africa a record US$ 5 billion (€ 3.4 billion) loan grant for the next three years to help the continent achieve its development goals. India has also offered an additional US$ 700 million (€ 482 million) for the establishment of new institutions and training programs in Africa and US$ 300 million (€ 207 million) support for the new Ethiopia – Djibouti railway line project. The Indian Prime Minister also suggested the establishment of several clusters including an India – Africa Food Processing Cluster and an India – Africa Institute of Agriculture and Rural Development. He finally proposed the creation of an India – Africa Virtual University, which will provide 10 000 new scholarships for African students.

## Energy

### Germany moves towards closing its nuclear power plants by 2021

A committee appointed by Chancellor Angela Merkel proposed that Germany should close all of its nuclear power plants by 2021. Nuclear energy currently meets nearly a quarter of Germany’s electricity needs, according to the Energy Ministry. The rest comes from coal supplies (42%), renewable sources like wind and solar energy (17%) and natural gas (14%). Interestingly, not even Japan, where a major nuclear scare occurred in March following an earthquake and tsunami, plans to abandon its reliance on nuclear power. Japan currently derives 30% of its electricity from nuclear power plants. Germany’s move away from nuclear energy, which partly reflects the strong influence of environmentalist groups in this country, is being closely watched by other European governments. Contrary to Germany, many nations in Central and Eastern Europe plan to develop or expand nuclear power production.

### IPCC projects that renewable sources could provide 77% of world’s energy by 2050

The experts from UN’s Intergovernmental Panel on Climate Change (IPCC) said that renewable sources could provide a majority of the world’s energy supplies by 2050 in their recent report. However, this projection is conditional on global governments’ dramatic increase of financial and political support for technologies such as wind and solar power. The report also stressed that the availability of renewable sources, like the wind and the sun, was virtually unlimited and could provide up to 77% of the world’s energy needs within the next 40 years. The report also pointed that all renewable sources used today, such as wind, solar, geothermal, hydropower, bioenergy and ocean energy, currently accounted for only about 13% of global energy supply. To scale this up to three quarters, large investments by governments and the private sector would be needed, amounting to US$ 5.1 trillion (€ 3.5 trillion) through 2020 and nearly US$ 7.2 trillion (€ 5 trillion) between 2021 and 2030.

### Bright prospects for wind power scale-up in the United States

Wind power is one of the fastest-growing sources of energy around the world. It is popular because it is abundant and clean, providing communities with their local source of electricity. In the United States, which has passed Germany and become the country producing the most wind power, the Department of Energy estimated that wind power could account for 20% of the nation’s electricity supply by 2030. Despite a prolonged recession and restricted credit markets, the wind power industry grew very strongly in the United States in 2009, adding 39% more capacity in comparison to a year earlier. The country is now very close to meeting 2% of its electricity needs from wind turbines. The American Wind Energy Association said the growth of wind power was helped by the federal stimulus package. The package extended a tax credit and provided other investment incentives for the industry.

### Winners of the annual Goldman Prizes announced

The annual Goldman Prizes, presented at a ceremony in San Francisco Opera House to six recipients this year, are awarded to activists who challenge those in power while either enhancing, or defending the environment. Each winner receives a stipend of US$ 150 000 (€ 103 305) stipend. The Goldman juries have been particularly prone to awarding those whose activities involve considerable risks. As an example, Wangari Maathai, the Kenyan founder of the Greenbelt Movement, has spurred the planting of tens of millions of trees across Africa. He won a Nobel Prize in 2004 – which was 13 years after receiving a Goldman Prize. This year’s winners include Ursula Sladek from Schönau, Germany, who created a small local power company, EWS, that rivalled the previous provider and which now provides electricity from renewable energy sources to her entire town and 110 000 other customers across Germany. Other recipients range from a Zimbabwe-based conservationist who worked to save the endangered black rhino, to a Texas man who fought refinery pollution in Port Arthur.

### Germany’s energy company among many to back out of India

Enercon of Germany is one of the world’s biggest makers of wind turbines. Recently, they announced a loss of its entire Indian subsidiary with annual sales of more than US$ 566 million (€ 390 million) after a dispute with a local partner and an encounter with Mumbai law enforcement authorities. They also claim that they have lost control of its patents in India, and fear that technology could be appropriated by their competitors in this big and growing market. The case has caused diplomatic tensions and clouded the image of India in Germany as a desirable investment market. Enercon is among many foreign companies and investors which have started to grow weary of the country’s widespread corruption, weak infrastructure and government limits on foreign investment in certain industries. Because of this and similar experiences, direct investment in India by foreign companies and investors fell by more than 31% in 2010, in comparison with the previous year.

## Peace and Human Rights

### United Nations declare internet access a basic human right

The UN declared that internet access should now be considered a human right. The Special Report states that the Internet is one of the most powerful instruments of the 21st century, because it helps increasing transparency, accessing information and facilitating active citizen participation in building democratic societies. However, given that access to basic necessities such as electricity remains difficult in many developing countries, the report states that universal access to the internet for all individuals worldwide cannot be achieved instantly, but it stretches the obligation for all countries to promote or facilitate the right to freedom of expression and the means necessary to exercise this, including the internet.

### UN reports on serious human rights breaches in more than 50 countries

A report, presented in early June 2011 to the UN Human Rights Council (UNHRC), documented serious violations of human rights in more than 50 countries. According to the UNHRC, some clear examples of these violations were the killings of demonstrators in Syria, Yemen and some other Arab countries. In addition, the report contains new evidence on alleged atrocities committed in the final stages of Sri Lanka’s civil war. The report concluded that there is a great need for transparent independent investigations into the human rights violations that have taken place in more than 50 countries.

### India and Pakistan agree to keep pushing for peace over Kashmir

According to the Associated Press, India’s foreign secretary and the next Ambassador to the United States, Nirupama Rao, said in June 2011 that her country would remain concerned about the threat of terrorism, but is committed to peace talks with Pakistan. Those talks have stalled since the 2008 terrorist attacks in Mumbai. Her comments came following a two-day gathering of the delegations of the two nations in Islamabad. The two countries, both with nuclear weapons, held their first formal talks on the disputed region of Kashmir since the Mumbai attacks. They have already fought three wars since their independence in 1947, two of them over Kashmir, which both nations claim in its entirety. The attacks in Mumbai left between 100 and 200 people dead and they have been blamed on Pakistani militants, who are suspected of building paramilitary forces and developing strongholds in Kashmir. Pakistan has denied that any state institutions were involved in any way with the attacks on Mumbai. The home secretaries met in New Delhi in March 2011 and agreed to set up a terrorism hotline and to cooperate on the Mumbai attack investigation, while the secretaries for commerce from both sides met in April 2011.

### US to pull out from Afghanistan, Europeans to follow swiftly

The President of the United States, Barack Obama, announced in June 2011 that a phased pullout of troops from Afghanistan will be set in motion, seeking to end this costly engagement. He currently plans to withdraw 10 000 troops by the end of 2011 and a further 23 000 by the end of the summer 2012. His announcement won immediate support from France’s President Nicolas Sarkozy, who promised to follow swiftly. After nearly a decade of fighting in Afghanistan, Obama’s withdrawal plan was welcomed by NATO allies. A number of other European nations which have contributed troops to the military operation against the Afghan Taliban insurgency said they would also initiate phased reductions. This mission has burdened state budgets and has been entirely against public opinion across much of Europe.

### US commission to watch over human rights in clinical trials

The United States Presidential Commission is a special commission, set up by President Barack Obama in 2009, which considers how best to protect the human rights of people who take part in clinical trials. This Commission was set up after the discovery that the US Public Health Service had conducted unethical research in Guatemala from 1946 to 1948, in which nearly 700 people were deliberately infected with syphilis and other sexually transmitted diseases. The trials were trying to show that penicillin could be used immediately after sex to prevent infection. Although an unethical experiment like the Guatemala trial is thought to be considerably less likely today, transparency, strict regulations and clearer guidelines are still necessary.

## Food, Water and Sanitation

### Rising food prices could threaten economic growth in Asia

In April 2011, the Asian Development Bank released a report in which it stated that sharp rises in food prices are a threat to economic growth in Asia. The bank made a gloomy prediction that this trend could soon push millions of people into extreme poverty. Food prices in Asia have increased at an average of about 10% in the first half of 2011, which could force more than 60 million people below the poverty income threshold of US$ 1.3 (€ 0.9) per person a day. Changyong Rhee, the chief economist of the bank, reminded that “...Asia is (still) home to two-thirds of the world’s poor.” Economic growth in China and India is blamed for pushing up prices, while the region’s population density and uneven income distribution make the lower social classes especially vulnerable to food prices growth. The poor in Asia typically spend nearly two-thirds of their income on food alone. The rise in prices of food and fuel leave Asia’s consumers with less income for other goods, while inflation could also prompt central banks to further raise interest rates. These factors would all work together to slow down economic growth.

### European Commission marks World Water Day by launching a new funding mechanism

The focus of World Water Day 2011, which is celebrated on 22 March each year, is ‘Water for cities – responding to the urban challenge’. The European Commission marked this day by announcing the launch of a pooling mechanism in the framework of the African, Caribbean, Pacific and European Union (ACP – EU) water facilities. Under this mechanism, the European Commission will provide 40 million Euros for grants from the European Development Fund (EDF) with further loans from the EU multilateral and bilateral □nance institutions. The scheme is expected to finance projects for access to water and sanitation services in African, Caribbean and Pacific countries. In most of the industrialized countries, nearly everyone has access to abundant supplies of safe and clean drinking water. However, in most low and middle income countries it is still not advisable to drink water from the tap.

### Sanitation Millennium Development Goal is badly off track

Despite all the progress in human development, 2.6 billion people, or about 40% of the total World’s population, still do not have access to proper sanitation. It is estimated that each year 1.5 million children of pre-school age die of diarrhoea caused by unsanitary conditions and poor hygiene. The UN’s Millennium Development Goal on expanding access to water and sanitation services by 2015 is very likely to be missed. Donors have increasingly avoided funding projects relevant to water and sanitation, and focused on health and education-related initiatives instead, according to research by the World Bank and Water Aid. The Guardian reports that women and girls will be among the hardest hit by this failure, quoting the World Bank’s report released in May 2011. Water Aid is also due to publish its new report this year, showing that water and sanitation programs accounted for about 8% of global financial aid in 1990, while between 2007 and 2009 they accounted for just over 5%. Julia Bucknall, the World Bank’s water chief, said that issues such as sanitation simply do not seem to be as attractive to donors as some other areas, particularly tackling specific diseases.

### A community-led approach to sanitation for low resource settings

Community-led total sanitation (CLTS) is gaining increasing attention as the Millennium Development Goal on sanitation is being missed. The traditional approach to hygiene has been education and subsidy. But in rural areas of low and middle income countries there have been many failed programmes, with toilets not being used or put to other purposes, or dismantled and materials used for other purposes. The cost of these failed development programmes runs into billions of dollars. CLTS does not use any standard design, hardware subsidy, teaching or any special measures. Communities are mobilised into analysing their own sanitation and waste behaviour, making their own participatory defecation and social maps, inspecting the areas of open defecation and analysing pathways to the mouth. The CLTS approach was pioneered in Bangladesh in 2000 by Kamal Kar, a development consultant from India. Since then he has been joined by many others to promote it, including Plan International, UNICEF, the Water and Sanitation Programme of the World Bank and Water Aid. The approach has now been adopted in more than 40 countries. It is usually driven by passionate champions, as many become committed once they experience the enhancement of their community’s human well-being. For women and girls it has helped to promote menstrual hygiene, self-respect, and the bodily well-being brought about by being able to defecate during daylight and in private.

### Fears over contamination of Japanese food exports

The United States’ Food and Drug Administration blocked imports from Japan’s radiation zone. It announced that it would avoid milk, vegetables and fruit from areas near the tsunami-smashed nuclear plant because of contamination fears. Other nations may follow with formal bans, while some private importers have stopped any shipments from Japan. Earlier, Japan had reported that above-safety radiation levels had been discovered in 11 types of vegetables from the area, in addition to milk and water. But the officials insisted that there was no danger to humans, and urged the world not to over-react. Tokyo authorities said water at a purification plant for the Japanese capital, with 13 million residents, had 210 becquerels of radioactive iodine, which was more than twice the level of safety for infants.

## Science and Technology

### Malaria vaccine trials move to Phase III with next generation vaccine already planned

It has been estimated that malaria still kills up to 800 000 people each year. GlaxoSmithKline and the PATH Malaria Vaccine Initiative recently started Phase III clinical trials on a developmental vaccine against malaria after Phase II testing proved effective. In the experiments completed in previous stages, the incidence of malaria was decreased by 53%. The potential to reduce episodes further was even larger if infants and young children were primarily targeted. Reuters reported further that even as the world’s first malaria vaccine moves closer to the market, GSK, PATH and Crucell have joined forces to test a next-generation vaccine against malaria. The new vaccine will be an amended version of the currently tested GSK vaccine. It will try to add an engineered common cold virus developed by Crucell to ‘prime’ the immune system to get a stronger response.

### Scientific publishers controversially tried to deprive poor countries from free access to journals

Lack of access to knowledge is widely accepted to be one of the main limitations to human development. In 2002, the World Health Organization launched the Health Inter-Network Access to Research Initiative (HINARI) project. Within this initiative, 137 publishers have provided content from 7000 journals free to local non-profit institutions in 105 eligible countries. Kimberly Parker, WHO’s HINARI programme manager, stated that 400 new journals were added to the network in 2010 alone. HINARI offers the opportunity of access to knowledge for the most resource-poor countries in the world. However, this programme seemed to be falling apart at one point during 2011, because big publishers began to withdraw from the scheme. Their decision has caused much debate and controversy. The HINARI program has recently been reviewed and an agreement seems to have been reached, in which publishers would continue to provide access until at least 2015.

### Researchers test needle-free, inhalable vaccine against measles

Sustained high vaccination coverage is critical to preventing deaths from measles. Despite the availability of a vaccine and its very high level of implementation globally, measles remains an important killer of children worldwide. The areas under most danger are deprived, less-developed regions where vaccination coverage is limited. A team of researchers, led by scientists from the Johns Hopkins Bloomberg School of Public Health and the University of Colorado, developed and successfully tested a dry powder containing live-attenuated measles vaccine that can be inhaled. The novel vaccine against measles was studied in rhesus macaques, and the results were published in January 2011 in the journal PNAS.

### The growing case for ‘open science’ and online raw data sharing

The value of routinely sharing the results of all clinical trials would be immense. Meta-analyses of the raw data from many clinical trials would have a potential to provide definitive answers on the effects of health interventions. The increasing use of electronic medical records in an anonymised format could provide high quality pharmacovigilance at unprecedented scale. However, a regime of open access to scientific data also poses many problematic questions. Because of the importance and timeliness of the issues, the UK’s Royal Society has established a Working Group to explore these questions, issues and challenges in great depth and to make recommendations about how they might be addressed. The Working Group is currently seeking evidence from scientists and from the public alike.

### China looking forward to becoming the new world leader in science and technology

Chinese Premier Wen Jiabao said in May 2011 that China “must develop powerful strength in science and technology and foster a large number of talented individuals in order to ‘gain the upper hand’ in international competition.” Addressing a plenary session of the National Congress of the China Association for Science and Technology (CAST), he stressed that the future of China relies on science and technology. He also said that China should improve the quality, performance and competitiveness of traditional industries through scientific and technological progress, suggesting that China should develop its own basic research and frontier research. The premier pledged that the government will provide long-term, stable financial assistance for basic and frontier research and set up a number of research centres, which will be based at high-level national research institutions and research-centred universities. He concluded that China should also gradually reform the systems of management, decision-making, appraisal, and personnel in the field of science and technology, so as to form a modern system in this sector that fits the country’s socialist market economy. He also pledged to firmly carry out the national strategy on intellectual rights, by stepping up efforts to protect them.

